# Symbiotic Characteristics of a New Indigenous *Sinorhizobium* Strain 98002 from *Medicago polymorpha* in Poyang Lake Wetland

**DOI:** 10.3390/microorganisms14102300

**Published:** 2026-10-09

**Authors:** Huajie Zhang, Yuhua Lu, Libing Liao, Yihong Hu, Yanling Chen, Tongjun Liang, Aiwen Zhong, Le Zhao, Deshui Yu

**Affiliations:** 1 School of Pharmacy, Henan University of Chinese Medicine, Zhengzhou 450046, China; 15514346353@163.com; 2 Lushan Botanical Garden, Jiangxi Province and Chinese Academy of Sciences, Jiujiang 332900, China; l2089232912@163.com (Y.L.);; 3 Jiangxi Provincial Key Laboratory of Wetland Plant Resources Conservation and Utilization, Jiujiang 332900, China

**Keywords:** *Sinorhizobium medicae*, *Medicago polymorpha*, Poyang Lake wetland, plant growth promotion, multilocus sequence analysis

## Abstract

*Medicago polymorpha* serves as an important green manure, forage, and edible wild vegetable. Symbiotic nitrogen fixation represents its primary nitrogen source; nevertheless, rhizobial strains that establish symbiotic interactions with this plant have rarely been isolated, particularly from distinctive wetland habitats. In this work, sinorhizobial strain 98002 was isolated from root nodules of wild *M. polymorpha* collected at Poyang Lake wetland. Scanning electron microscopy (SEM) was applied to observe its cellular morphological features, and molecular identification combining 16S rRNA sequencing and six concatenated housekeeping gene multilocus sequence analysis (MLSA) confirmed the isolate as *Sinorhizobium medicae*. Antibiotic susceptibility tests demonstrated that strain 98002 was fully sensitive to seven common antibiotics. Host inoculation assays further verified that this indigenous strain could develop pink nodules on *M. polymorpha* roots and remarkably improve host biomass accumulation under nitrogen-deficient conditions. This indigenous rhizobial resource serves as promising germplasm for sustainable *M. polymorpha* cultivation in the Poyang Lake basin.

## 1. Introduction

Nitrogen is the most critical macronutrient for plant growth and frequently acts as the primary limiting factor in global agricultural systems [1]. The global nitrogen cycling network, predominantly driven by diverse microbial communities, is essential for maintaining biosphere productivity and health [2]. Over the past few decades, the rapid intensification of modern agriculture has led to unprecedented reliance on synthetic nitrogen fertilizers to meet the food demands of a growing global population [3]. However, systemic overuse of these chemical fertilizers has triggered a cascade of severe environmental crises, including widespread soil acidification, catastrophic eutrophication of inland and coastal waters, and a marked increase in emissions of potent greenhouse gases such as nitrous oxide [4,5,6]. Optimizing biological nitrogen fixation (BNF) is therefore widely recognized as a vital strategy for transitioning to a sustainable and low-carbon agricultural model [7,8,9].

The symbiotic association established between leguminous plants and specialized soil bacteria, known as rhizobia, ultimately drives the reduction of atmospheric nitrogen to ammonia, a bioavailable nitrogen source that can be directly absorbed and metabolized by host legumes [10,11]. Recent genomic studies of *Medicago* species have provided deep insights into these symbioses, highlighting the intricate requirements for optimal nodulation [12,13,14,15]. This efficiency bottleneck is mainly attributed to the inconsistent performance of commercial rhizobia inoculants across diverse and complex soil environments [16]. Exotic laboratory strains, while highly effective under sterile conditions, often lack environmental resilience and struggle to establish robust populations in complex field soils [17]. Moreover, they frequently fail to successfully colonize host root nodules when facing intense competition from indigenous microbial communities [18].

A strategic solution to this problem lies in the identification and utilization of indigenous rhizobia strains [19,20,21]. Unlike exotic inoculants, native rhizobia have undergone long-term co-evolution with local host plant genotypes and developed specific physiological adaptations perfectly suited to the physicochemical properties of their native soils [19,20,21,22]. These indigenous populations possess superior ecological fitness, enabling them to withstand localized abiotic stresses, such as extreme pH fluctuations, nutrient deficiencies, and extreme temperatures [23]. Crucially, native strains can maintain high nodule occupancy even under intense microbial competition [24]. Therefore, mining native rhizobia resources pre-adapted to specific regional conditions is considered a top priority for developing high-efficiency, site-specific biofertilizers [25].

The Poyang Lake wetland in southern China represents a unique and highly dynamic system for exploring adaptive microbial diversity [25]. As China’s largest freshwater lake, the wetland is characterized by extreme spatiotemporal hydrological fluctuations and dramatic seasonal cycles of submergence and exposure [25]. These intense water–land transitions exert strong selective pressures on soil microbial communities, potentially driving the evolution of specialized native rhizobia lineages equipped with distinct stress tolerance mechanisms and enhanced symbiotic performance.

*Medicago polymorpha*, a leguminous species, is widely distributed in the Poyang Lake wetland. It serves not only as an excellent forage crop but also as a leafy vegetable; more importantly, this species is a high-quality green manure [13,26]. As a forage crop, it is characterized by rapid growth, high biomass accumulation, and excellent palatability; its crude protein content at early flowering reaches approximately 17.8–22.2%, comparable to that of tetraploid alfalfa (*M. sativa*), rendering it a high-quality feed source for cattle, sheep, and other livestock [13]. Furthermore, in the Yangtze River basin and surrounding regions, *M. polymorpha* has a centuries-long history of cultivation as a traditional leafy vegetable, prized for its tender texture, distinctive flavor, and rich nutritional profile [13]. Beyond its macronutrient content, *M. polymorpha* is a notable source of micronutrients and bioactive phytochemicals. When compared with eight wild edible plants, *M. polymorpha* showed the highest total phenolic content and displayed the strongest 2,2-diphenyl-1-picrylhydrazyl (DPPH) radical-scavenging activity and reducing power [26]. However, despite its widespread distribution, research on the symbiotic rhizobia diversity associated with *M. polymorpha* specifically in the Poyang Lake wetland remains notably insufficient.

To fill this critical knowledge gap, we isolated and identified indigenous rhizobia from root nodules of *M. polymorpha* in the Poyang Lake wetland. Using the 16S rRNA gene and multilocus sequence analysis (MLSA) based on the six core housekeeping genes, we precisely determined its taxonomic status. Combined with phenotypic characterization and host reinoculation experiments, our results provide a valuable microbial resource for boosting the productivity of *M. polymorpha* in southern China.

## 2. Materials and Methods

### 2.1. Plant Material

*M. polymorpha* plants with root nodules and their seeds were collected from the bank of Poyang Lake at a latitude of 29°37′45″ N and a longitude of 116°8′20″ E. Mature and plump seeds were collected after ripening and used as the test plant material.

*M. polymorpha* is a widespread wild edible plant of the Poyang Lake wetland in China, and this species is not listed in any national or international threatened plant inventories. The sampling site was the lakeshore of Poyang Lake, which is outside protected nature reserve areas, so no special collection permit or license was required for sampling. Only a small quantity of plant root nodules and seeds were collected, and the sampling activities exerted no adverse impact on local wild plant populations and their natural habitats.

Formal taxonomic identification of *M. polymorpha* was completed with reference to voucher specimen NAS00619965 preserved in the Herbarium of the Institute of Botany, Jiangsu Province, the Chinese Academy of Sciences. This reference voucher was originally collected by the Lushan Botanical Garden. No additional independent voucher specimens of the sampled materials were collected or deposited in public herbaria for this study.

### 2.2. Isolation and Purification of Bacterial Strain

Fresh root nodules were collected from *M. polymorpha* growing on the bank of Poyang Lake. The nodules were first rinsed with sterile water to remove surface soil, then disinfected with 75% (*v*/*v*) ethanol for 30 s and rinsed three times with sterile water. Subsequently, the nodules were disinfected with 5% (*v*/*v*) sodium hypochlorite solution for 5 min and rinsed with sterile water another 5 times. The final rinse water was collected and spread onto various solid media and incubated at 28 °C for ~5 d to confirm complete surface sterilization of nodules. The disinfected nodules were blotted dry with sterile filter paper and crushed with a pipette tip in a 2.0 mL EP tube; 400 μL of sterile water was added, and the mixture was mixed thoroughly by repeated pipetting. The suspension obtained from the homogenized root nodules was then inoculated onto modified yeast–mannitol agar (M-YMA) plates containing the following components per liter of distilled water, adjusted to pH 6.8: D-mannitol, 10 g; yeast extract, 1 g; L-sodium glutamate, 0.5 g; K_2_HPO_4_, 0.5 g; MgSO_4_, 0.1 g; NaCl, 0.05 g; CaCl_2_, 0.04 g; FeCl_3_, 0.04 g; and agar, 15–20 g. The plates were incubated at 28 °C for 2–3 days, and single colonies were picked for further purification.

For strain purification, the picked single colonies were streaked onto M-YMA solid medium plates and incubated at 28 °C until new single colonies formed. The streak purification process was repeated three times to obtain pure cultures of strain 98002. The strain was preserved in glycerol stock at −80 °C for long-term storage and used for subsequent experiments. Additionally, the purified strain has been deposited in the China General Microbiological Culture Collection Center (CGMCC) with the accession number CGMCC No. 37337.

### 2.3. Morphological and Physiological Assays

Strain 98002 was incubated at 28 °C for 2–3 days on M-YMA solid medium plates, and colony characteristics such as morphology, size, color, margin, and elevation were observed. For the antibiotic resistance test, the seed culture was streaked on TY plates (1 L containing 5 g tryptone, 3 g yeast extract, and 0.87 g CaCl_2_·2H_2_O, pH 7.0) without antibiotics (Ctrl) or with one of the following antibiotics at different concentrations: kanamycin (Kan), streptomycin (Str), rifampicin (Rif), carbenicillin (Car), tetracycline (TC), neomycin (Neo), and hygromycin (Hgy). The plates were incubated at 28 °C for 2–3 days, and growth was recorded.

For microscopic morphology analysis, pure cultures of strain 98002 were collected and handled with caution to avoid mechanical damage. Bacterial cells were gently rinsed with PBS to remove residual medium, followed by immediate fixation with electron microscopy fixative in the dark at room temperature for 30 min. The fixed samples were subsequently sent to Wuhan Servicebio Technology Co., Ltd. (Wuhan, China) for scanning electron microscopy (SEM) analysis.

### 2.4. DNA Extraction and Molecular Identification

Genomic DNA was extracted from pure cultures of strain 98002 using a bacterial genomic DNA extraction kit (Sangon Biotech, Shanghai, China) following the manufacturer’s instructions. The extracted genomic DNA was stored at −20 °C until further use.

For determining the species affiliation of strain 98002, the 16S rRNA sequence [27] and six housekeeping genes (*recA*, *atpD*, *glnII*, *rpoB*, *gyrB* and *dnaK*) [19] were amplified by polymerase chain reaction (PCR) using the extracted genomic DNA as the template. The PCR system (30 μL) consisted of 1 μL of DNA template, 1 μL of forward primer (10 μM), 1 μL of reverse primer (10 μM), 15 μL of 2× PCR Mix (KAPA-HiFi), and 12 μL of ddH_2_O. The PCR amplification program was set as follows: initial denaturation at 95 °C for 8 min; 30 cycles of denaturation at 98 °C for 15 s, annealing at 60 °C for 20 s, and extension at 72 °C for 1.5 min; final extension at 72 °C for 5 min; and holding at 12 °C. The primers used for each gene amplification are detailed in Supplementary Table S1. After PCR amplification, the PCR products were loaded onto a 1% (*w*/*v*) agarose gel (TransGen Biotech, Beijing, China) and electrophoresed at 120 V for 20 min. The electrophoresis results were visualized using a gel imaging system. PCR products with clear bands of the expected target size were purified using the TransGen Gel Extraction Kit (TransGen Biotech, Beijing, China) following the manufacturer’s instructions. Purified PCR products were then submitted to Sangon Biotech Co., Ltd. (Shanghai, China) for Sanger sequencing.

The obtained 16S rRNA gene sequence of strain 98002 was subjected to BLAST analysis in the GenBank database via the NCBI web BLAST service (https://blast.ncbi.nlm.nih.gov/Blast.cgi accessed on 5 May 2025), to identify closely related strains. The sequences of housekeeping genes from closely related strains were downloaded from GenBank (Supplementary Table S2). A phylogenetic tree was constructed using MEGA7 based on 16S rRNA sequences to clarify the taxonomic status of strain 98002. Further sequences of six housekeeping genes (*recA*, *atpD*, *glnII*, *rpoB*, *dnaK*, and *gyrB*) from strain 98002 and reference *Sinorhizobium* strains were used to build a phylogenetic tree independently using MEGA7. Multilocus sequence analysis (MLSA) based on the six housekeeping genes (concatenated in the above order) was also performed to further confirm the species information of strain 98002.

### 2.5. Plant Inoculation

The *M. polymorpha* seeds were surface-disinfected as follows: the seeds were first soaked in 75% (*v*/*v*) ethanol for 30 s and rinsed thoroughly with sterile water two to three times. Subsequently, the seeds were immersed in 5% (*v*/*v*) sodium hypochlorite solution for 5–8 min, followed by 5–6 rinses with sterile water to completely remove residual disinfectants. Finally, the disinfected seeds were soaked in sterile water and germinated on a horizontal shaker at room temperature with a rotation speed of 60 rpm for 1 day. After the seeds germinated and showed white radicles, they were sown into a sterile substrate (nutrient soil:vermiculite = 3:1, *v*/*v*) with 5 seeds per pot. One week after sowing, the seedlings were carefully removed from the sterile substrate, rinsed with deionized water to remove residual substrate, and then transferred into plastic square pots with an inner side length of 6.5 cm and a height of approximately 7.5 cm. Each pot was filled with green zeolite to a depth of approximately 6 cm, and three uniform seedlings were transplanted per pot. The zeolite was thoroughly rinsed and autoclaved before use.

One night before inoculation, strain 98002 and the incompatible control rhizobial strain *Sinorhizobium meliloti* Sm1021 [20] were activated in TY medium and cultured in a constant-temperature incubator at 28 °C with shaking at 180 rpm until reaching the logarithmic growth phase (OD_600_ ≈ 0.8). The bacterial culture was then inoculated into fresh TY medium at a ratio of 1:100 (*v*/*v*) for expansion culture. The expanded bacterial suspension was centrifuged at 4000 rpm for 10 min, washed twice with 1/2 BNM medium [28], and diluted to OD_600_ = 0.05 to prepare the inoculum. For each treatment, 10 mL of the corresponding inoculum was irrigated into the substrate of each pot. An uninoculated control group (Ctrl) was established in parallel, in which each pot received 10 mL of sterile 1/2 BNM medium without bacterial cells; all other conditions were identical to those of the inoculated treatments. The inoculated plants were cultured in an artificial climate chamber (Kesheng, Ningbo, China) under the following conditions: day/night temperature 24/20 °C, light intensity 15,000 lux, photoperiod 12 h light/12 h dark, and relative humidity 60–70%. During the cultivation period, sterile water was regularly irrigated to maintain the substrate moisture. Phenotypic analysis was conducted at 21 days post-inoculation. Whole plant fresh weight and shoot fresh weight per plant were individually measured for phenotypic quantification. All quantitative data were subjected to one-way analysis of variance (ANOVA), and statistical analyses and visualization of biomass-related phenotypic differences were performed using the online tool ImageGP 2 (https://www.bic.ac.cn/BIC/#/, accessed on 1 September 2026) [29].

### 2.6. Nucleotide Sequence Deposition

The 16S rRNA and six housekeeping gene (*recA*, *atpD*, *glnII*, *rpoB*, *dnaK*, *gyrB*) sequences of strain 98002 generated in this work have been deposited in GenBase [30] at the National Genomics Data Center [31], Beijing Institute of Genomics, Chinese Academy of Sciences/China National Center for Bioinformation (https://ngdc.cncb.ac.cn/genbaseaccessed on 7 July 2026). Their corresponding accession numbers are listed below: 16S rRNA (C_AA474794), *recA* (C_AA474795), *atpD* (C_AA474796), *glnII* (C_AA474797), *rpoB* (C_AA474798), *dnaK* (C_AA474799), *gyrB* (C_AA474800).

## 3. Results

### 3.1. Isolation and Morphological Characteristics of Rhizobia Strain 98002

**Figure 1:**
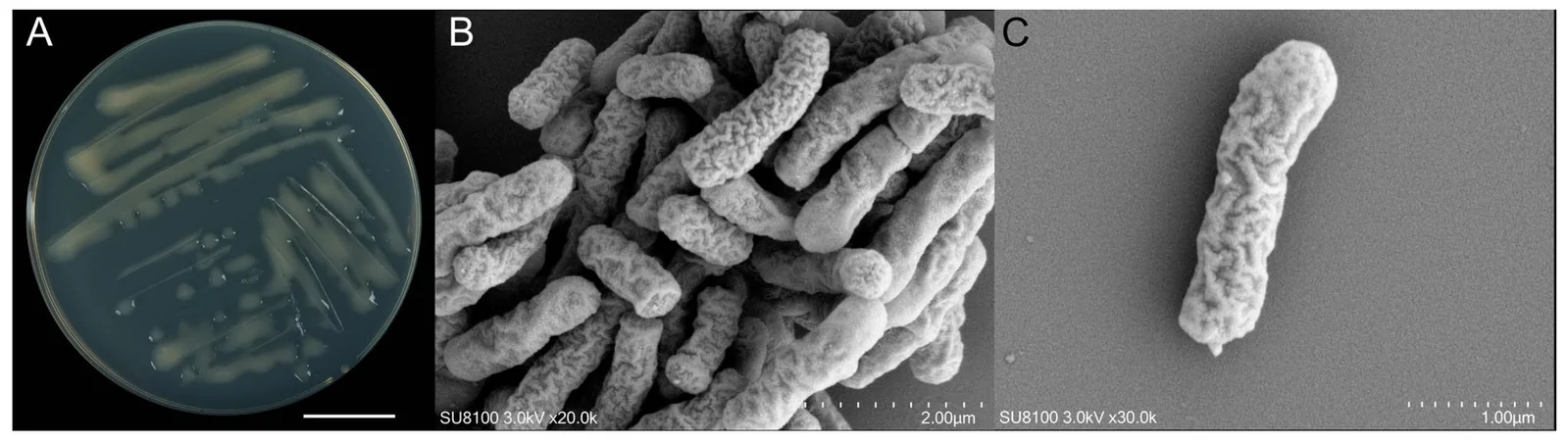
Morphological characteristics of Rhizobium strain 98002 isolated from root nodules of *M. polymorpha*. (**A**) Colonial morphology of strain 98002 grown on modified yeast–mannitol agar (M-YMA) medium at 28 °C. Bar = 2 cm. (**B**) Scanning electron microscopy (SEM) image of strain 98002 at 20.0 k magnification, scale bar = 2.00 μm. (**C**) High-magnification SEM image of strain 98002 at 30.0 k magnification, scale bar = 1.00 μm.

A bacterial strain, designated 98002, was successfully isolated and purified from root nodules of *M. polymorpha* collected in the Poyang Lake wetland. The strain exhibited typical rhizobial growth characteristics on modified yeast–mannitol agar (M-YMA) medium. After incubation at 28 °C, colonies were white, circular, convex, moist, and viscous, consistent with the general morphological description of rhizobia (Figure 1A). Scanning electron microscopy (SEM) observations of the purified strain 98002 were performed using a Hitachi SU8100 instrument at magnifications of 20.0k× and 30.0k× to characterize its cellular morphology (Figure 1B,C). The bacterium was rod-shaped, with an average length of approximately 2.0 μm and a diameter of about 0.6 μm, excluding flagella. The cell surface appeared slightly textured under high magnification.

### 3.2. Strain 98002 Is Susceptible to Various Antibiotics

**Figure 2:**
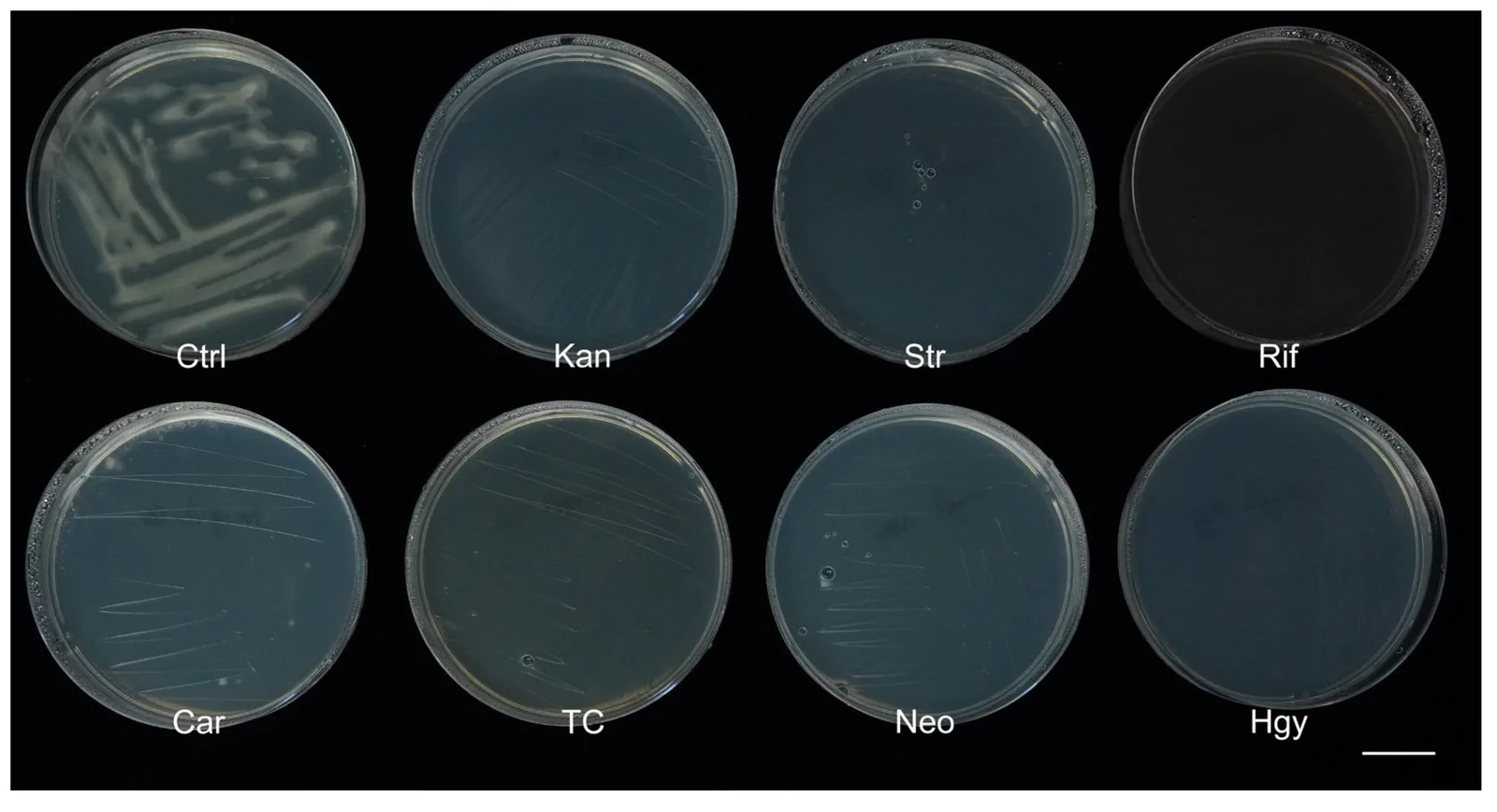
Antibiotic susceptibility test of Rhizobium strain 98002. The strain was streaked on TY solid medium supplemented with different antibiotics. Ctrl: control (no antibiotics); Kan: kanamycin (50 μg/mL); Str: streptomycin (50 μg/mL); Rif: rifampicin (50 μg/mL); Car: carbenicillin (50 μg/mL); TC: tetracycline (10 μg/mL); Neo: neomycin (50 μg/mL); Hgy: hygromycin (50 μg/mL). No bacterial growth was observed on any antibiotic-containing plates, while the control plate showed normal growth. Bar = 2 cm.

The antibiotic resistance profile of strain 98002 was evaluated by culturing the isolate on TY solid medium supplemented with different antibiotics. Robust bacterial growth was observed only on the antibiotic-free control (Ctrl) plate, where typical rhizobial colonies developed normally (Figure 2). In contrast, no visible colonies were detected on any of the antibiotic-containing media after incubation, indicating that strain 98002 was sensitive to all tested antibiotics (Figure 2).

Further, strain 98002 was cultured on TY solid medium supplemented with a dilution gradient of each antibiotic (2–50 µg/mL). Complete growth inhibition was achieved at the following concentrations: Kan ≥ 25 µg/mL, Str ≥ 25 µg/mL, Rif ≥ 10 µg/mL, Car ≥ 50 µg/mL, TC ≥ 5 µg/mL, Neo ≥ 2 µg/mL, and Hyg ≥ 10 µg/mL (Supplementary Table S3). These results confirmed that strain 98002 is highly susceptible to all seven antibiotics tested in this study, especially to neomycin; 98002 showed complete inhibition at the lowest concentration tested (2 µg/mL). This broad sensitivity profile provides a key phenotypic marker for the strain and will support future genetic manipulation studies requiring appropriate selection markers.

### 3.3. Strain 98002 Can Form Symbiotic Nodules with M. polymorpha and Promote Plant Growth

To evaluate the symbiotic performance of strain 98002, *M. polymorpha* seedlings were either inoculated with strain 98002 or the incompatible strain *Sinorhizobium meliloti* Sm1021 or left uninoculated (Ctrl). All plants were grown under nitrogen-deficient conditions for 21 days. Sm1021 served as the incompatible control because it is unable to establish an effective nitrogen-fixing symbiosis with *M. polymorpha* [20].

**Figure 3:**
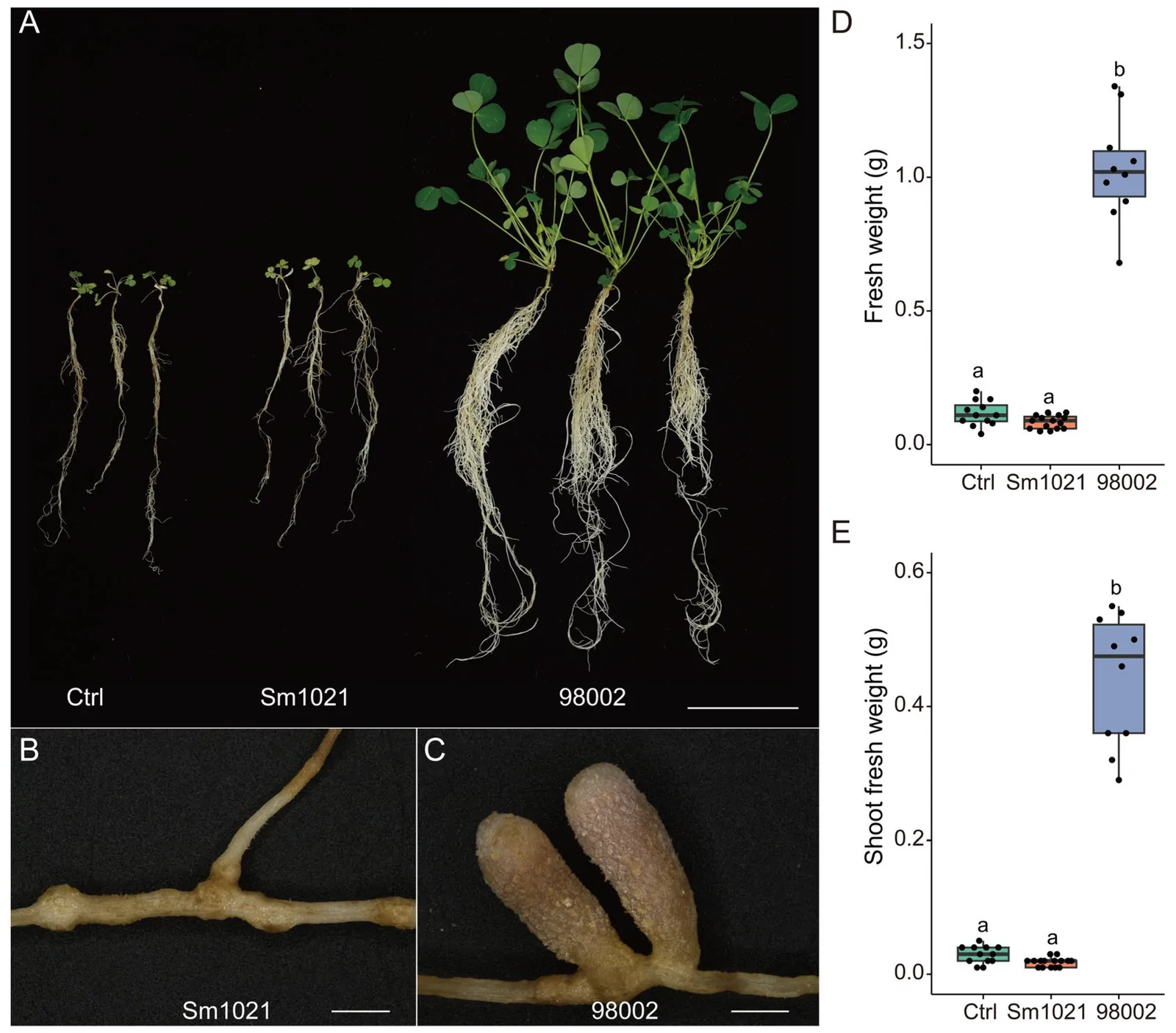
Symbiotic performance of Rhizobium strain 98002 on *M. polymorpha*. (**A**) Growth phenotypes of *M. polymorpha* plants inoculated with strain 98002 (right), incompatible strain Sm1021 (middle), and uninoculated control plants (Ctrl, left) at 21 days post-inoculation. Scale bar = 5 cm. (**B**) A close-up view of a representative root from an Sm1021-inoculated plant. Scale bar = 1 mm. (**C**) A close-up view of a representative root nodule induced by strain 98002. Scale bar = 1 mm. (**D**) Whole-plant fresh weight per plant of *M. polymorpha* in the three treatments at 21 days post-inoculation. (**E**) Shoot fresh weight per plant of *M. polymorpha* in the three treatments at 21 days post-inoculation. Data in (**D**,**E**) are presented as box plots with individual biological replicates overlaid (*n* ≥ 10). Different lowercase letters (a, b) indicate statistically significant differences between treatments (*p* < 0.05). The experiment was repeated at least three times independently, and similar results were obtained in each replicate.

Marked differences in plant vigor were observed among the three treatments (Figure 3A). Plants inoculated with strain 98002 exhibited significantly enhanced growth, including taller shoots, larger and darker green trifoliate leaves, and a more extensive root system (98002), compared with both Sm1021-inoculated and uninoculated plants (Sm1021 and Ctrl, respectively). In contrast, the Sm1021-inoculated and uninoculated plants displayed clear symptoms of nitrogen deficiency, characterized by chlorotic leaves and severely stunted growth, exhibiting indistinguishable phenotypes from each other (Figure 3A).

Microscopic examination of the root systems revealed distinct, pinkish nodules (10–18 nodules per plant (mean = 13.6)) on the roots of plants inoculated with strain 98002 (Figure 3C). These nodules were cylindrical in shape and exhibited the typical morphological features of indeterminate nodules, consistent with those formed by the model legume *M. truncatula* [32]. The pink coloration, attributable to the accumulation of leghemoglobin, may indicate that these nodules were metabolically active and potentially capable of supporting nitrogen fixation. For plants inoculated with Sm1021, only small, white and rudimentary nodules (Figure 3B) were detected, similarly to a previous report [20].

Quantitative analysis of plant biomass further confirmed the growth-promoting effect of strain 98002. At 21 days post-inoculation, the whole-plant fresh weight of 98002-inoculated plants was significantly higher than that of the Sm1021 and Ctrl groups’ plants, approximately 10-fold higher than that of the two control groups (Figure 3D), and the shoot fresh weight showed a similarly dramatic increase (Figure 3E).

Collectively, these results demonstrate that strain 98002 is capable of establishing an effective symbiotic relationship with *M. polymorpha*, forming indeterminate nodules, and significantly promoting host plant growth under nitrogen-deficient conditions.

### 3.4. Strain 98002 Is a Strain of Sinorhizobium medicae

To clarify the taxonomic status of strain 98002, we adopted a tiered phylogenetic analysis strategy: First, the 16S rRNA gene was used to determine its approximate phylogenetic placement. Second, individual phylogenetic reconstructions were performed for each of the six housekeeping genes (*recA*, *atpD*, *glnII*, *rpoB*, *dnaK*, and *gyrB*). Finally, MLSA based on these six housekeeping genes was conducted to obtain a robust, species-level phylogenetic resolution.

The amplicons of strain 98002 were subjected to BLAST analysis in GenBank, and representative strains of the same genus were selected as reference strains, including the widely used model strain *S. medicae* WSM419, as well as *S. meliloti* strains ABS7, Sm1021, and Sm2011 (Supplementary Table S2). The target gene sequences of reference strains were retrieved from their corresponding whole-genome sequences; the sequences of the reference strains and strain 98002 were then imported into MEGA 7 software, aligned using MUSCLE, and phylogenetic trees were constructed using the Maximum Likelihood (ML) method.

**Figure 4:**
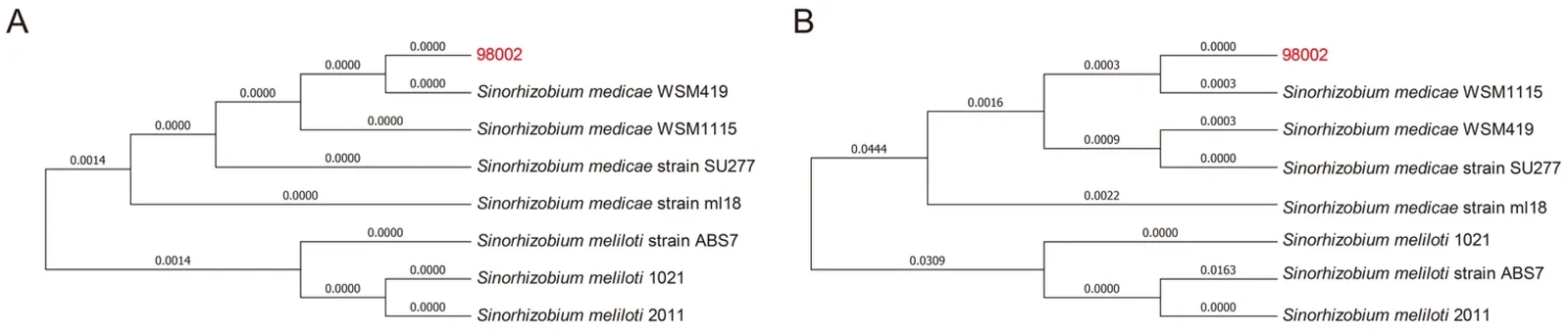
Phylogenetic placement of strain 98002 within the genus *Sinorhizobium*. (**A**) 16S rRNA gene-based phylogenetic tree. (**B**) MLSA tree based on concatenated sequences of six housekeeping genes: *recA*, *atpD*, *glnII*, *rpoB*, *dnaK*, and *gyrB*. Strain 98002 is highlighted in red. The trees were constructed using the Maximum Likelihood method, and evolutionary distances (substitutions per site) are shown next to the branches.

The 16S rRNA gene-based phylogeny suggested that strain 98002 formed a tight monophyletic cluster with reference strains of *S. medicae* (including WSM419, WSM1115, SU277 and ml18), with extremely low pairwise distances (0.0000 substitutions per site) (Figure 4A), and the 16S rRNA sequence showed 100% similarity to this species (Supplementary Figure S1). The *S. medicae* clade was clearly separated from the sister species *S. meliloti* (strains ABS7, 1021, and 2011), with a mean interspecific distance of 0.0014 substitutions per site (Figure 4A). A similar result was obtained when a phylogenetic tree was built using six housekeeping genes (*recA*, *atpD*, *glnII*, *rpoB*, *dnaK*, and *gyrB*) (Supplementary Figure S2).

Consistent with the individual genes’ data (16S rRNA and six housekeeping genes), the MLSA phylogeny based on concatenated sequences of all six housekeeping genes (Figure 4B, Supplementary Table S2, and Supplementary Data) confirmed the placement of strain 98002 within the *S. medicae* lineage. Strain 98002 grouped most closely with *S. medicae* WSM1115 (0.0003 substitutions per site), forming a well-supported subclade together with WSM419 and SU277. The *S. medicae* clade was clearly distinct from the *S. meliloti* clade, with a much higher interspecific divergence (0.0444 substitutions per site at the root node), highlighting the discriminatory power of MLSA for resolving species boundaries in this genus.

Collectively, both the individual genes (16S rRNA and six housekeeping genes) and MLSA phylogenies consistently demonstrated that strain 98002 belongs to the species *S. medicae*.

## 4. Discussion

In this study, we isolated and characterized an indigenous rhizobial strain, designated 98002, from root nodules of wild *M. polymorpha* growing in the Poyang Lake wetland. Through a polyphasic taxonomic approach combining 16S rRNA sequences and multilocus sequence analysis (MLSA) of six housekeeping genes, we identified strain 98002 as *Sinorhizobium medicae*. Host reinoculation assays demonstrated that this native strain forms functional, pink indeterminate nodules on *M. polymorpha* roots and promotes host biomass accumulation under nitrogen-deficient conditions.

*M. polymorpha* is a multifunctional annual legume that has a centuries-long history of cultivation in the Yangtze River basin as a traditional leafy vegetable, prized for its tender texture and distinctive flavor [13]. The forage, green-manure, vegetable, and functional-food values establish *M. polymorpha* as an exceptionally versatile plant for low-input, diversified, and sustainable agriculture, particularly when reducing dependence on inorganic nitrogen fertilizers is a global priority [33].

Despite this multifaceted value, research on *M. polymorpha* and its symbiotic rhizobia partners remains markedly underdeveloped relative to the model legume *M. truncatula* and the major forage crop *M. sativa*. Two gaps are especially pertinent to the present study. First, the rhizobia diversity associated with *M. polymorpha* across China is poorly characterized: to date, only a single systematic survey has been published, focusing on purple soils in Yunnan [21], while rhizobia resources from the wetland ecosystems of the middle and lower Yangtze River basin—including the Poyang Lake wetland—remain entirely unexplored. Second, *M. polymorpha* exhibits unusually high symbiotic specificity: it fails to form effective symbioses with a broad range of naturalized soil rhizobia, and commercial inoculants are largely ineffective on this host [20,34]. Critically, among *Sinorhizobium* strains, only *S. medicae* induces well-developed, pink, nitrogen-fixing nodules on *M. polymorpha*, whereas *S. meliloti*, including the model strain Sm1021 used as the incompatible control in the present study (Figure 3A), forms only small, white, and rudimentary nodules (Figure 3B) [20]. The same study also revealed a striking ecological correlation: *S. medicae* was predominantly recovered from medics adapted to moderately acidic soils (including *M. polymorpha*, *M. arabica*, and *M. murex*), whereas *S. meliloti* was almost exclusively associated with alkaline/neutral-soil species such as *M. littoralis* and *M. tornata* [20]. This host–symbiont–soil pH coupling indicates that the choice of symbiotic partner in nature is strongly shaped by environmental adaptation rather than by host identity alone. This ecological pattern is fully consistent with the pedoclimatic context of our sampling site: the Poyang Lake basin is dominated by acidic red soils that have undergone further acidification in recent decades [35,36,37]. The recovery of strain 98002, identified as *S. medicae*, from *M. polymorpha* root nodules in this acidic wetland habitat therefore corroborates the acid-soil enrichment of *S. medicae* documented in Mediterranean soils and identifies this species as the ecologically expected, pre-adapted symbiont for *M. polymorpha* across acid-soil regions of southern China.

Accurate taxonomic delineation of rhizobia at the species level remains a persistent challenge, particularly within the Rhizobiaceae family, where 16S rRNA gene sequences alone often fail to resolve diverged lineages recently due to the slow evolutionary rate and intragenomic heterogeneity of this locus [38,39]. In our analysis, the 16S rRNA gene phylogeny placed strain 98002 within the *S. medicae* clade with 100% sequence similarity to reference strains, yet the extremely low interspecific divergence between *S. medicae* and its sister species *S. meliloti* (0.0014 substitutions per site) underscored the limited resolving power of this marker (Figure 4A and Supplementary Figure S1). To overcome this limitation, we employed MLSA based on six concatenated housekeeping genes (*recA*, *atpD*, *glnII*, *rpoB*, *dnaK*, and *gyrB*), an approach widely recognized as the gold standard for species-level identification in *Sinorhizobium* [38]. The MLSA phylogeny not only confirmed the affiliation of strain 98002 with *S. medicae* but also revealed its closest relationship with *S. medicae* WSM1115, with a markedly higher interspecific divergence from *S. meliloti* (0.0444 substitutions per site) that clearly resolved the species boundary (Figure 4B and Supplementary Figure S2). Notably, a recent survey of *Sinorhizobium* populations associated with *M. polymorpha* in purple soils of southern China found that *S. medicae* was the dominant symbiont in *M. polymorpha* nodules [21], lending further support to the notion that *S. medicae* is the primary symbiotic partner of *M. polymorpha* across diverse soil types in southern China. Our isolate from the Poyang Lake wetland thus extends the known geographic distribution of the *S. medicae*–*M. polymorpha* symbiosis to subtropical lake wetland habitats.

The symbiotic performance of *S. medicae* 98002 on its native host *M. polymorpha* was evaluated under controlled, nitrogen-deficient conditions. At 21 days post-inoculation, plants inoculated with *S. medicae* 98002 exhibited dramatically enhanced growth compared with controls, as evidenced by significantly greater whole-plant fresh weight and shoot fresh weight when compared with plants inoculated with the incompatible strain Sm1021 and uninoculated plants (Figure 3D,E). The 98002-inoculated plants developed cylindrical, pink indeterminate nodules (Figure 3C), whose coloration reflects the accumulation of leghemoglobin-a hallmark of active nitrogen-fixing symbiosis [32]. These results demonstrate that *S. medicae* 98002 is a symbiont capable of satisfying the nitrogen requirements of *M. polymorpha* under low-nitrogen conditions; direct quantification of nitrogenase activity and plant nitrogen content will be required in future work to further evaluate the nitrogen-fixing contribution of strain 98002. The remarkable biomass promotion conferred by *S. medicae* 98002 (Figure 3) can be primarily ascribed to the home-field advantage characteristic of indigenous rhizobial strains originating from the local wetland habitat. Because they are already perfectly adapted to local soil microbiomes, they exhibit vastly superior physiological fitness in their native soils. The Poyang Lake wetland represents a uniquely dynamic and challenging wetland ecosystem, leading to extreme wet-dry cycles [25]. We hypothesize that strain 98002 has evolved directly under these rigorous ecological conditions and may have acquired essential genetic material from other indigenous soil microbes [40]. Furthermore, leguminous host plants have evolved highly sophisticated “host sanction” mechanisms, by which the plant selectively rewards high-efficiency strains with more photosynthetic carbon resources [40].

Notably, strain 98002 exhibited complete sensitivity to all seven tested antibiotics (kanamycin, streptomycin, rifampicin, carbenicillin, neomycin, hygromycin, and tetracycline) (Figure 2 and Supplementary Table S3). Broad antibiotic susceptibility is often characteristic of bacteria originating from relatively pristine, unpolluted environments that have not been subjected to sustained anthropogenic antibiotic selection pressure. From an applied perspective, this trait carries both advantages and implications. On one hand, the absence of antibiotic resistance genes simplifies genetic manipulation, as any of the commonly used resistance markers can be employed for mutant construction and complementation assays. On the other hand, and perhaps more importantly from a biosafety standpoint, the lack of acquired antibiotic resistance determinants reduces the risk of horizontal gene transfer of resistance traits to indigenous soil or clinical bacteria following field application, making strain 98002 a safer candidate for large-scale environmental release [22]. Furthermore, it has been argued that strains not burdened by the maintenance and expression of antibiotic resistance cassettes may allocate a greater proportion of their metabolic energy to nitrogen fixation, potentially enhancing symbiotic efficiency [41].

Despite the promising results presented in this study, several important limitations and corresponding future research directions of strain 98002 remain to be systematically addressed. Its agronomic efficacy, persistence, and competitive nodulation ability against indigenous rhizobia under fluctuating field environments still require validation through multi-site, multi-season field trials. While MLSA provides robust taxonomic identification, whole-genome sequencing would offer far deeper insights into the complete architecture of its symbiotic gene clusters, as well as accessory genomic regions potentially linked to environmental stress tolerance and rhizosphere competence. Finally, the current host range assessment is restricted to *M. polymorpha*, and systematic inoculation tests on economically important congeners such as *M. sativa* and the model legume *M. truncatula* are still needed to determine whether this strain can establish effective symbiosis across a broader set of hosts. These ongoing efforts will support the future development of this strain as an effective inoculant for sustainable legume production.

## 5. Conclusions

In conclusion, this study reports the isolation and comprehensive characterization of *S. medicae* strain 98002 from root nodules of wild *M. polymorpha* in the Poyang Lake wetland. Polyphasic taxonomic analysis using 16S rRNA sequencing, six housekeeping genes, and MLSA based on the six housekeeping genes firmly established its species identity as *S. medicae*. Host inoculation assays confirmed its ability to form metabolically active and potentially functional nitrogen-fixing nodules and significantly enhance *M. polymorpha* biomass under nitrogen-deficient conditions. Its broad antibiotic susceptibility further supports its suitability as a safe and tractable microbial resource. Collectively, these findings identify strain 98002 as a promising indigenous biofertilizer candidate for sustainable *M. polymorpha* production in the Poyang Lake basin and adjacent subtropical wetland regions.

## Data Availability

The datasets generated during the current study are available in the GenBase repository of the National Genomics Data Center, with accession numbers as follows: 16S rRNA (C_AA474794), *recA* (C_AA474795), *atpD* (C_AA474796), *glnII* (C_AA474797), *rpoB* (C_AA474798), *dnaK* (C_AA474799), *gyrB* (C_AA474800), which is publicly accessible at https://ngdc.cncb.ac.cn/genbase (accessed on 7 July 2026).

## Supplementary Materials

The following supporting information can be downloaded at https://www.mdpi.com/article/10.3390/microorganisms14102300/s1. Supplementary Table S1. Sequences of primers used in this study; Supplementary Table S2. GenBank accession numbers of genomic sequences for strains used in this study; Supplementary Table S3. Growth inhibition of strain 98002 by different antibiotics at a concentration gradient; Supplementary Figure S1. Multiple sequence alignment of 16S rRNA sequences of strain 98002 and reference strains; Supplementary Figure S2. Maximum-likelihood phylogenetic trees constructed from six housekeeping genes. Phylogenetic trees based on single-gene sequences of *recA* (A), *atpD* (B), *glnII* (C), *rpoB* (D), *dnaK* (E), and *gyrB* (F), respectively. Strain 98002 is highlighted in red font. All trees were built using the Maximum-Likelihood method in MEGA7. Branch lengths represent evolutionary distances (substitutions per site). Reference strains include multiple *Sinorhizobium medicae* strains (WSM419, WSM1115, SU277, ml18) and *Sinorhizobium meliloti* strains (ABS7, 1021, 2011); Supplementary Data S1. Detailed sequence information of housekeeping genes utilized in this study.
