# Supplementary material for: Symbiotic Characteristics of a New Indigenous Sinorhizobium Strain 98002 from Medicago polymorpha in Poyang Lake Wetland
Source: Microorganisms. 2026 Oct 9;14(10):2300. doi: 10.3390/microorganisms14102300 (PMC13707402; doi:10.3390/microorganisms14102300)
Supplement: Supplementary file 1 [file microorganisms-14-02300-s001.zip › Supplementary FigureS2.pdf]

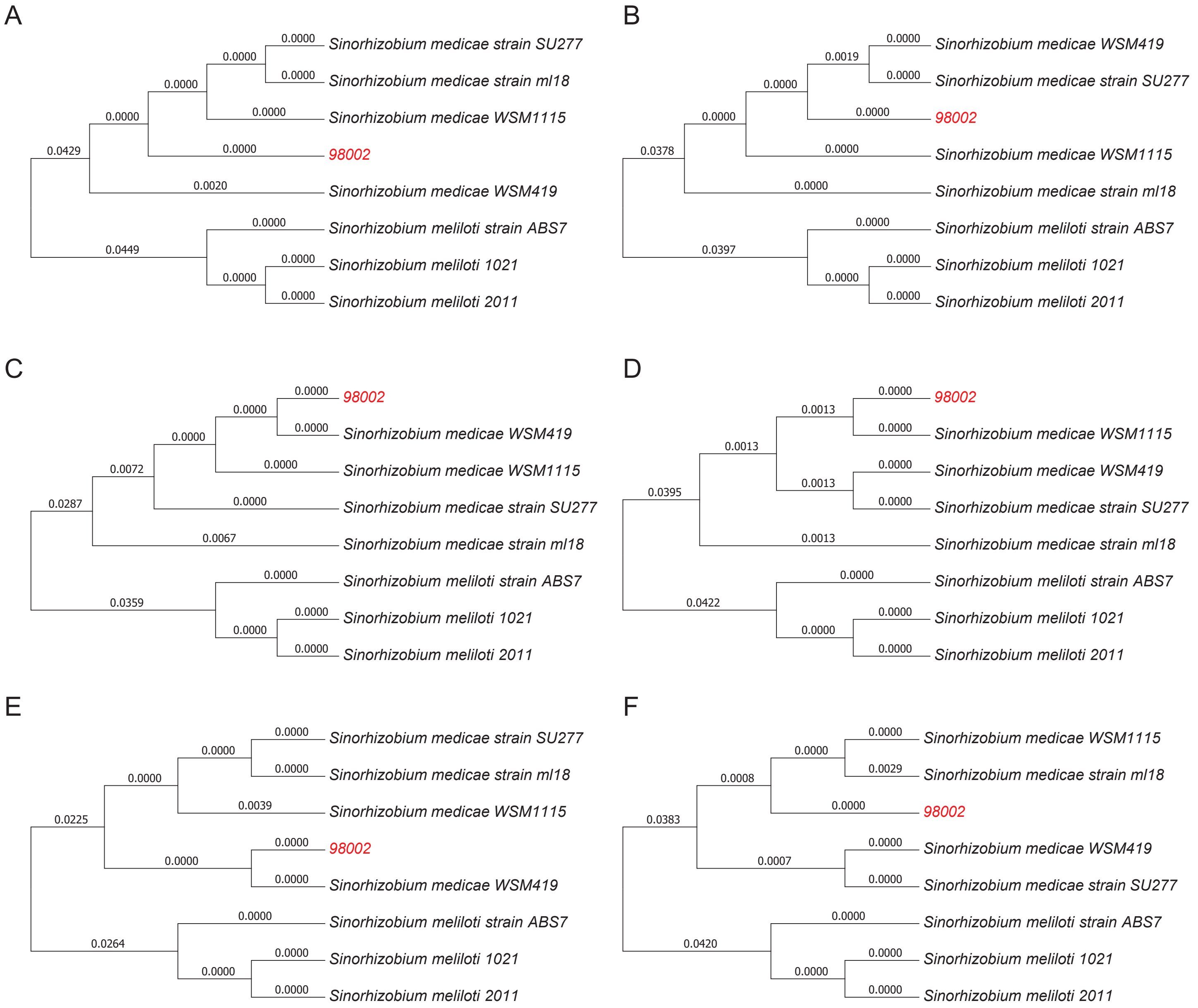

A
Sinorhizobium medicae strain SU277
Sinorhizobium medicae strain ml18
Sinorhizobium medicae WSM1115
98002
Sinorhizobium medicae WSM419
Sinorhizobium meliloti strain ABS7
Sinorhizobium meliloti 1021
Sinorhizobium meliloti 2011
B
Sinorhizobium medicae WSM419
Sinorhizobium medicae strain SU277
98002
Sinorhizobium medicae WSM1115
Sinorhizobium medicae strain ml18
Sinorhizobium meliloti strain ABS7
Sinorhizobium meliloti 1021
Sinorhizobium meliloti 2011
C
98002
Sinorhizobium medicae WSM419
Sinorhizobium medicae WSM1115
Sinorhizobium medicae strain SU277
Sinorhizobium medicae strain ml18
Sinorhizobium meliloti strain ABS7
Sinorhizobium meliloti 1021
Sinorhizobium meliloti 2011
D
98002
Sinorhizobium medicae WSM1115
Sinorhizobium medicae WSM419
Sinorhizobium medicae strain SU277
Sinorhizobium medicae strain ml18
Sinorhizobium meliloti strain ABS7
Sinorhizobium meliloti 1021
Sinorhizobium meliloti 2011
E
Sinorhizobium medicae strain SU277
Sinorhizobium medicae strain ml18
Sinorhizobium medicae WSM1115
98002
Sinorhizobium medicae WSM419
Sinorhizobium meliloti strain ABS7
Sinorhizobium meliloti 1021
Sinorhizobium meliloti 2011
F
Sinorhizobium medicae WSM1115
Sinorhizobium medicae strain ml18
98002
Sinorhizobium medicae WSM419
Sinorhizobium medicae strain SU277
Sinorhizobium meliloti strain ABS7
Sinorhizobium meliloti 1021
Sinorhizobium meliloti 2011
