# Supplementary material for: Symbiotic Characteristics of a New Indigenous Sinorhizobium Strain 98002 from Medicago polymorpha in Poyang Lake Wetland
Source: Microorganisms. 2026 Oct 9;14(10):2300. doi: 10.3390/microorganisms14102300 (PMC13707402; doi:10.3390/microorganisms14102300)
Supplement: Supplementary file 1 [file microorganisms-14-02300-s001.zip › Supplementary tableS1-S3.pdf]

Supplementary Table S1. Sequences of primers used in this study

Amplicon	Forward and Reverse Primers（5'-3'）
16S	27F：AGAGTTTGATCCTGGCTCAG<br>1492R：TACGGCTACCTTGTTACGACTT
recA	41F：CGTTCGGCAAGGGATCGATCATG<br>640R：CATGCGGATCTGGTTGATGAAG
atpD	255F：GCTSGGCCGCATCMTSAACGTC<br>782R：GCCGACACTTCMGAACCNGCCTG
glnII	12F：YAAGCTCGAGTACATYTGGCT<br>689R：TGCATGCCSGAGCCGTTCCCA
gyrB	343F：TTCGACCAGAAYTCCTAYAAGG<br>1043R：AGCTTGTCCTTSGTCTGCG
rpoB	454F：ATCGTCTCGCAGATGCACCG<br>1364R：TCGATGTCGTCGATYTCGCC
dnaK	1466F：AAGGARCANCAGATCCGCATCCA<br>1777R：TASATSGCCTSRCCRAGCTTCAT

Supplementary Table S2. GenBank accession numbers of genomic sequences for strains used in this study

Strain	Genome sequence	Plasmid sequence
S. medicae WSM419	CP000738.1	CP000739.1
S. medicae WSM1115	CP088109.1	CP088110.1
S. medicae strain SU277	CP104147.1	CP104148.1
S. medicae strain ml18	CP140924.1	CP140926.1
S. meliloti strain ABS7	CP073681.1	CP073680.1
S. meliloti 1021	AL591688.1	AL591985.1
S. meliloti 2011	CP004140.1	CP004139.1

Supplementary Table S3. Growth inhibition of strain 98002 by different antibiotics at a concentration gradient.

Antibiotic	Concentration (μg/mL)				
	50	25	10	5	2
Kanamycin	-	-	+	+	N.D.
Streptomycin	-	-	+	+	N.D.
Rifampicin	-	-	-	+	N.D.
Carbenicillin	-	+	+	+	N.D.
Tetracycline	N.D.	N.D.	-	-	+
Neomycin	-	-	-	-	-
Hygromycin	-	-	-	+	N.D.

Bacterial growth was recorded on TY solid medium after incubation at 28°C for 72 h. Symbols: −, complete inhibition of growth (no colonies); +, no inhibition (normal colony growth); N.D., not done.
